# Accidental Intravenous Administration of Milk in a Newborn: A Case Report on Management Strategies and Prevention Protocols

**DOI:** 10.7759/cureus.73371

**Published:** 2024-11-10

**Authors:** Sultana Baydoun, Issa Awaida, Naufal Naufal

**Affiliations:** 1 Department of Neonatology, Lebanese University Faculty of Medicine, Beirut, LBN; 2 Department of Pediatrics, Lebanese University Faculty of Medicine, Beirut, LBN

**Keywords:** accidental administration, iv administration, neonatal intensive care unit (nicu), pediatric case report, urgent pediatric care

## Abstract

In the following article, we present a case report detailing a medical error wherein a post-term baby boy, admitted to our neonatal intensive care unit due to respiratory distress and hypoglycemia, inadvertently received enteral formula via umbilical venous catheter over approximately one hour. Our report encompasses the hospital course, management strategies, and the resultant outcome. Immediately following the incident, the infant exhibited symptoms of respiratory distress, mottled skin, hypotension, and thrombocytopenia.

Prompt intervention entailed administering supportive care, closely monitoring vital signs, particularly blood pressure, with the administration of vasopressors, and initiating broad-spectrum antibiotic therapy. Fortunately, the infant responded well to treatment and exhibited no discernible long-term adverse effects. Subsequent to the incident, thorough investigations were conducted to ascertain its root cause. Furthermore, measures were implemented to enhance medical staff education regarding such occurrences and to develop preventive protocols.

## Introduction

Ensuring patient safety is paramount in medical practice, compelling healthcare teams to strive for the prevention of medical errors to avoid potentially fatal complications and life-threatening events. Enteral feeding, defined as the administration of nutrition through a nasogastric tube or stoma directly into the gastrointestinal tract, represents a critical aspect of patient care. However, inadvertent administration of enteral feeding solutions through an intravenous (IV) line can precipitate a spectrum of complications and potentially fatal outcomes, including systemic infections, allergic reactions, organ damage, shock, thrombosis, and mortality.

The imperative to differentiate between enteral and IV routes becomes especially salient in clinical scenarios necessitating the use of multiple tubes, such as peripheral and central lines, feeding tubes (nasogastric or stomal), or peritoneal catheters. Given the gravity of potential complications, healthcare professionals must adhere rigorously to established guidelines and protocols governing the administration of enteral nutrition and IV medications or total parenteral nutrition (TPN). This adherence is essential not only to mitigate the risk of life-threatening events but also to safeguard the well-being of patients under their care.

In this case report, we present a detailed analysis of an incident involving the inadvertent administration of milk via an IV line in a pediatric patient, highlighting the ensuing complications, management strategies, and preventive measures implemented to enhance patient safety.

## Case presentation

We present a case of a 43-week post-term newborn boy, delivered via urgent cesarean section due to gestational hypertension in a G2P1A1 mother. The infant exhibited Apgar scores of 6 and 7 at one and five minutes, respectively, and was admitted to our neonatal intensive care unit (NICU) due to respiratory distress and desaturation to 85%, followed by hypoglycemia shortly after birth.

Upon admission, a full sepsis workup was conducted, and empiric antibiotic therapy with ampicillin and gentamicin was initiated. The infant required nasal cannula oxygen support, and hypoglycemia was corrected with a dextrose infusion. Due to challenges encountered during intravenous access, an umbilical venous catheter was inserted.

Subsequent investigations revealed negative blood and cerebrospinal fluid cultures, though C-reactive protein (CRP) levels were elevated, peaking at 33.2 mg/dL on day four of life. On day six of life, and during a night shift, the nursing staff observed signs of respiratory distress, including tachypnea, desaturation, tachycardia (heart rate of 180 bpm), and mottled skin. Upon further investigations, it was discovered that enteral feeding had been inadvertently infused into the bloodstream via the umbilical venous catheter, resulting in a bolus of approximately 4 cc over one hour.

The incident prompted an immediate response that included removal of the umbilical venous catheter, insertion of a new catheter, and securing the airway through intubation. To manage the newborn’s evolving condition, the patient was kept nil per os, and antibiotic therapy was adjusted, with fluconazole added to meropenem to cover a potential fungal infection. Intravenous hydrocortisone was also administered to control persistent hypoglycemia, despite a high glucose infusion rate (GIR) through the second umbilical venous catheter.

Laboratory findings confirmed the development of disseminated intravascular coagulation (DIC), evidenced by thrombocytopenia (platelet count of 17,000/μL), prolonged prothrombin time (PT), international normalized ratio (INR), and activated partial thromboplastin time (aPTT) (Table 1). Fresh frozen plasma (FFP) was administered every eight hours based on the patient’s coagulation profile and clinical response to address DIC while balancing the benefits of coagulation support against risks such as allergic reactions, volume overload, and transfusion-related acute lung injury (TRALI). Linezolid was substituted for vancomycin after a positive tip culture for gram-positive *Staphylococcus haemolyticus*, resistant to vancomycin, while blood cultures remained negative.

**Table 1:** Laboratory findings of the patient.

Parameter	Value	Reference range
C-reactive protein (CRP)	33.2 mg/dL	<1 mg/dL
Platelet count	17,000/μL	150,000-450,000/μL
Prothrombin time (PT)	Prolonged	11-13.5 seconds
International normalized ratio (INR)	Prolonged	0.8-1.1
Activated partial thromboplastin time (aPTT)	Prolonged	30-40 seconds
Blood culture	Negative	Negative
Cerebrospinal fluid culture	Negative	Negative

Additional laboratory data provided further insights. The peripheral white blood cell (WBC) count was elevated at 18,000/μL, and liver function tests showed an increased serum glutamic-pyruvic transaminase (SGPT) level of 150 U/L. Blood gas analysis indicated moderate acidosis, while kidney function and electrolytes remained within normal limits. A complete blood count revealed leukocytosis, anemia, and a stable hemoglobin level. Diagnostic imaging, including brain and cardiac echocardiography, was unremarkable aside from a small patent foramen ovale (PFO). After five days of intensive management, the infant stabilized and was discharged home without apparent lasting injuries.

## Discussion

The inadvertent administration of enteral feeding through an IV line in clinical settings, though rare, is a significant patient safety concern, particularly in NICUs. Such an error occurred in the case of our post-term neonate, admitted for hypoglycemia and respiratory distress, where milk was mistakenly infused through a central IV line. This type of error often arises in complex cases involving multiple lines, such as stomas, enteral feeding tubes, and central lines, that increase the potential for line mix-ups. Factors like identical pump usage for both feeding and IV infusions, syringe mislabeling, and insufficient training further contribute to the risk. Although our NICU unit has a low incidence of neonatal sepsis, the occurrence of such errors underscores the need for proactive safety strategies.

Errors of this nature can lead to a range of consequences, from mild allergic reactions to severe, potentially fatal complications such as systemic infections, fluid overload, organ damage, venous thrombosis, and even death [1]. Enteral formulas, which are non-sterile and hypertonic, are not designed for direct bloodstream administration, making their IV introduction hazardous. The hypertonic composition of these formulas can precipitate electrolyte imbalances, increasing the risk of heart failure and other complications, while microbial contamination can result in severe sepsis, septic shock, and even DIC with multi-organ dysfunction. Previous reports, including that by Ryan et al., documented fatalities associated with similar incidents, resulting in three fatalities out of eight cases [2]. Sindi et al. reported an 11-month-old patient who experienced sepsis and acute respiratory distress following the IV infusion of formula milk, though the patient recovered within 48 hours [3]. Another review from 1972 described a case involving IV administration of breast milk, leading to respiratory distress and seizures, but the patient survived without lasting adverse effects after supportive management. These cases highlight the critical need for stringent line verification protocols and error-prevention measures in NICU settings.

Medical errors occur at a significantly higher rate in NICUs compared to adult care settings, largely due to the physiological immaturity of neonates and the intricate nature of the care they require. A study from Benha University Hospital found that 97% of neonates experienced at least one medical error during their NICU stay, with a total of 3,819 errors recorded among 148 patients [4]. Medication errors are a particularly pressing concern, accounting for about 10.55% of total errors, with dispensing errors being the most frequent. A study from Iran echoes this concern, revealing that 74.8% of patients had encountered at least one medication error, with antibiotics being the most commonly involved drugs [5]. Such findings highlight the unique vulnerabilities of neonates in the NICU, underscoring the need for targeted interventions and robust safety protocols.

Indeed, errors in NICUs often stem from complex and interrelated factors, including staffing shortages, environmental conditions, and equipment deficiencies. Studies show that errors are more prevalent during evening and night shifts, reflecting how factors like staff fatigue and reduced personnel levels impact patient safety [6]. Additionally, issues with essential medical equipment can contribute to adverse events, as deficiencies or malfunctioning devices may hinder timely and accurate care. These findings support the need for a systems-based approach to error prevention, recognizing that most medical errors result from systemic flaws rather than individual negligence. This perspective aligns with the Swiss cheese model of accident causation, which emphasizes addressing system-level issues, such as workflow design, equipment functionality, and protocol compliance, over focusing on individual culpability [7]. Statements that imply blame for errors, such as suggesting staff “crowding,” “speed,” or “lack of attention,” can inadvertently create a culture of fear rather than learning. Instead, a non-punitive, systems-oriented approach fosters an environment that encourages error reporting and continuous improvement.

Recognizing the critical nature of the event, our team initiated a root cause analysis (RCA) to identify contributing factors and assess the adequacy of existing safety protocols, including double-check processes and line verification, which are central to enteral and IV administration. Indeed, structured RCA and incident reporting are critical tools for identifying the contributing factors to errors, including workplace environment, shift fatigue, and the adequacy of safety protocols [8]. Insights from RCA inform staffing decisions, protocol refinements, and targeted training initiatives, which together reduce the likelihood of future errors. Healthcare provider training should prioritize not only treatment strategies but also education on medication and nutrition errors, as well as comprehensive line management skills.

Moreover, research suggests that innovative technologies offer promising ways to mitigate human error, adding important safeguards without burdening NICU staff. For example, electronic medical records (EMR) streamline provider communication and reduce medication errors; color-coded syringes and tubing systems distinguish between different lines, helping to prevent administration errors; and radio-frequency identification (RFID)-enabled monitoring systems reinforce workflow clarity and enhance patient safety [6]. Preventive measures, such as tracing tubing origins before connecting, proper labeling, and utilizing distinct pumps for feeding and IV infusions, are essential safeguards [9-11]. Some centers have even employed methylene blue in enteral formulas as a visual aid to prevent mix-ups while reducing nurse-to-patient ratios and ensuring specialized neonatal nurses are part of the care team, further strengthening these efforts.

Fostering a culture of safety requires both an organizational commitment to prioritize patient safety and a dedication to continuous training and education. When staff feel encouraged to report potential hazards without fear of retribution, they are more likely to engage in proactive error prevention. Enhancing nurse-to-patient ratios to align with global standards and investigating automated systems, such as computerized physician order entry (CPOE), could further reduce the chance of human error in medication management. Attention to the precise control of enteral feeding administration, ensuring correct amounts, compositions, and rates, is imperative to support safe outcomes. Collectively, these efforts create a safer, more resilient environment for one of the most vulnerable patient populations, neonates, enabling NICUs to effectively mitigate the risk of critical incidents and enhance patient safety.

## Conclusions

The inadvertent intravenous administration of enteral formulas, though rare, can result in significant adverse effects, requiring immediate and effective management. Prompt intervention with supportive therapies, broad-spectrum antibiotics, and collaborative multidisciplinary teamwork is crucial in mitigating the impact of such medical errors. Laboratory findings, such as elevated CRP, thrombocytopenia, and coagulopathy, highlight the severity of the error and underscore the importance of timely recognition and intervention.

To prevent these incidents, healthcare facilities should prioritize comprehensive education for staff and caregivers, emphasizing the importance of proper administration protocols. In addition, implementing meticulous labeling, clear segregation of enteral and intravenous systems (such as color-coded tubing and connectors), and a two-person verification policy can further reduce the risk of errors. Conducting an RCA after each incident would also be beneficial in identifying and addressing potential system failures. These preventive strategies, grounded in the clinical data and lessons from this case, are critical in enhancing patient safety and reducing the risk of similar errors in the future.
